# Metabolic and Epigenetic Action Mechanisms of Antidiabetic Medicinal Plants

**DOI:** 10.1155/2019/3583067

**Published:** 2019-05-05

**Authors:** Siba Shanak, Bashar Saad, Hilal Zaid

**Affiliations:** ^1^Faculty of Sciences, Arab American University Palestine, P.O Box 240, Jenin, State of Palestine; ^2^Qasemi Research Center- Al-Qasemi Academy, P.O Box 124, Baqa El-Gharbia 30100, Israel

## Abstract

Diabetes is a predominant metabolic disease nowadays due to the off-beam lifestyle of diet and reduced physical activity. Complications of the illness include the gene-environment interactions and the downstream genetic and epigenetic consequences, e.g., cardiovascular diseases, tumor progression, retinopathy, nephropathy, neuropathy, polydipsia, polyphagia, polyuria, and weight loss. This review sheds the light on the mechanistic insights of antidiabetic medicinal plants in targeting key organs and tissues involved in regulating blood glucose homeostasis including the pancreas, liver, muscles, adipose tissues, and glucose absorption in the intestine. Diabetes is also involved in modulating major epigenetic pathways such as DNA methylation and histone modification. In this respect, we will discuss the phytochemicals as current and future epigenetic drugs in the treatment of diabetes. In addition, several proteins are common targets for the treatment of diabetes. Some phytochemicals are expected to directly interact with these targets. We lastly uncover modeling studies that predict such plausible interactions. In conclusion, this review article presents the mechanistic insight of phytochemicals in the treatment of diabetes by combining both the cellular systems biology and molecular modeling.

## 1. Background

Diabetes mellitus (DM) is a metabolic ailment resulting from insulin resistance or the reduced secretion of insulin. Insulin disorders result in distorted carbohydrate, fat and protein metabolism, and the increased levels of serum glucose. Uncontrolled hyperglycemia may end up damaging blood vessels and causing macrovascular (atherosclerotic) and microvascular (retinopathy and nephropathy) disorders [1]. Besides, HDL/LDL ratio in the serum decreases [2]. It is accepted that diabetes is a result of the imbalanced regulation at the genetic and epigenetic levels. Pancreatic *β*-cell differentiation is controlled by several genes such as GLP1 receptor, PAX4, and PDX. These genes are regulated at the epigenetic level. Additionally, some factors that are involved in insulin resistance, such as NF-kB, osteopontin, and Toll-like receptors, are epigenetically regulated.

Healthy lifestyle can be used to alleviate hyperglycemia. Nonetheless, this might fail to treat diabetes in a large number of cases. In this regard, medications should be introduced. Thus, understanding the different molecular and cellular mechanisms of action for the glycemic control helps in planning and introducing active chemicals for the treatment of diabetes. Several pharmaceutical drugs present in the market have limited actions and many side effects, e.g., biguanides and sulphonylurea. The scientific introduction of medicinal plants is a good alternative for the treatment of diabetes [3]. Indeed, active phytochemical should be screened and validated to test for their efficacy and toxicity. Herein, we summarize the mechanisms of action for the antidiabetic activity of drugs, with the emphasis on plants and their active phytochemicals. Moreover, the inhibition of epigenetic marks associated with diabetes is detailed. Herein, light is shed on medicinal plants and active ingredients that target diabetes via epigenetic mechanisms. These epigenetic drugs, or “epidrugs”, target DNA methyltransferases, histone-modifying enzymes, e.g., histone deacetylases, histone acetyltransferases, protein arginine methyltransferases, histone methyltransferases, and histone demethylases.

Moreover, phytochemical “lead compounds” that target diabetes are currently screened via methods of* in silico *drug design. In this review, we introduce protein targets for the antidiabetic drugs. Additionally, we show that several plants and the derived phytochemicals were put under the microscope by methods of computer-aided drug design. These plants include dried leaves of green tea, pomegranate, complex phenols in olive oil, linalool of* C. sativum*,* Papaver somniferum*-derived papaverine, and components of ginger, as well as* Euphorbia thymifolia* Linn. extracts. Hence, we concentrate on the cellular and molecular levels in drug design. It is thus worth mentioning that phytochemical screening for the treatment of diabetes is transferred into a new epoch.

## 2. Introduction

Diabetes mellitus (DM) is a metabolic disorder, where insulin resistance or the reduced levels of secreted insulin cause hyperglycemia [4]. Insulin is a key anabolic hormone, involved in signalling cascades that regulate complex carbohydrates, fats, and proteins synthesis. According to the World Health Organization (WHO), more than 422 million people worldwide were diabetic in 2014, and this number is expected to double in 2040. The prevalence of diabetes is the highest in the Middle East (13.7% in 2014), where the number of diabetic patients reached 43 million in 2014 [5].

Based on the etiology of DM, two main types of diabetes are known. Type I diabetes has very low prevalence. In most cases of this form, autoimmune mechanisms target the pancreatic *β*-cells to destruction, what drives the necessity for insulin replacement therapy [4, 6]. Type II diabetes is more predominant, and it results from insulin resistance in target tissues or the shortage in insulin secretion [4].

**Figure 1:**
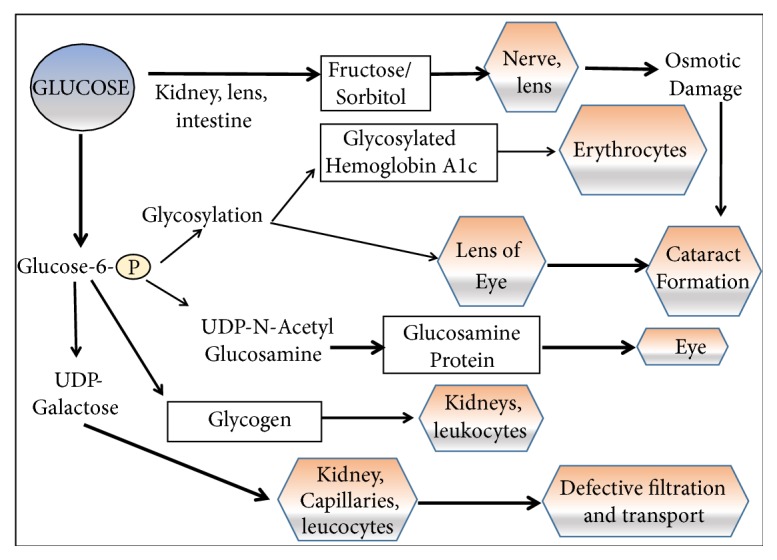
*Consequences of hyperglycemia.* As a result of osmotic imbalance and many others, hyperglycemia contributes to damage in several organs, e.g., eye, kidney, leukocytes, and capillaries.  Figure 1 is reproduced from Saad et al. (2017) ([under the Creative Commons Attribution License/public domain)” [14].

Since insulin signalling serves as a metabolic hub, carbohydrate, fat, and protein metabolism are drastically distorted after the increase in blood glucose levels. Fasting glycemia, postprandial glycemia, and haemoglobin A1C levels are elevated to ≥7mM, ≥11mM, and 6.5%, respectively [7]. Uncontrolled hyperglycemia for prolonged periods results in the destruction of blood vessels supplying the body organs, with the consequence of heart, eyes, kidneys, and nerves system damage. As a result, macrovascular (atherosclerotic) and microvascular (retinopathy and nephropathy) disorders follow. These complications are the leading causes of mortality in diabetic patients [1]. Moreover, levels of serum LDL increase, whereas the serum levels of HDL decrease [2]. Additional complications include blurred vision, polyuria, polydipsia, polyphagia, and weight loss [8].  Figure 1 introduces the causal relationship between hyperglycemia and the resulting damage in different organ systems. Extracellular hyperglycemia ends up in oxidative stress due to the oxidation and glycation reactions between reducing sugars and proteins [9]. Consequently, glycoxidation products, such as N*ε*-(carboxymethyl)-lysine and N*ε*-(carboxymethyl)-hydroxylysine, as well as free radicals, accumulate in tissue collagen of diabetic patients, causing metabolic stress, tissue damage, and cell death [10]. Among the consequences of tissue damage are changes in eye refraction [11], infiltration difficulties in the kidney [12], and others.

It is widely appreciated that genetic and epigenetic factors predispose to diabetes. Major genes that control *β*-cell differentiation, such as GLP1 receptor, PAX4, and PDX1, are epigenetically regulated. Epigenetics also induce insulin resistance through proinflammatory effects on some factors, as NF-kB, osteopontin, and Toll-like receptors [13].

To avoid hyperglycemia or alleviate the symptoms, preventive strategies through nonpharmacological approaches can be followed. Healthy diet, exercise, and weight loss can adjust glucose serum levels and improve normal glucose metabolism. When lifestyle change fails to treat diabetes, medications become a necessity. In type I DM, therapeutic insulin replacement is introduced. Additionally, pancreatic islets can be transplanted [16]. Drugs were on the other hand designed to target type II DM, with variant modes of actions. Some drugs induce an increased production and secretion of insulin in the *β*-pancreatic cells. Other drugs promote insulin sensitivity in the target tissues. Liver tissue is responsible for buffering blood glucose and secreting glucose to the bloodstream to retain glucose homeostasis. Thus, hepatic enzymes involved in gluconeogenesis and glycogenolysis are inhibited via insulin signalling. On the other hand, skeletal muscles and adipose tissues are stimulated to increase glucose uptake [17]. Additionally, abnormal lipolysis induces hyperglycemia, reduced insulin secretion, and/or glucose uptake. Thus, some drugs that target diabetes are designed to inhibit lipolysis. Abnormal lipolysis also results in lipotoxicity, with the accumulation of toxic lipid metabolites (ceramide, diacylglycerol, and fatty acyl CoA) in adipocytes, muscles, liver, and the pancreas. Cardiovascular diseases are a major consequence of lipolysis [18]. Such complications cannot be treated via the aforementioned mechanisms and need agents that regulate the vascular homeostasis to relieve the injury and reduce the inflammation [19]. The most direct route for glycemic control is via the inhibition of glucose absorption in the intestinal walls. Thus enzymes that digest complex polysaccharides into simpler absorbable forms are inhibited [20] via, for example, alpha-glucosidase competitive inhibitors [21]. Major complications of diabetes include a plausible increase in the inflammatory responses. Thus, anti-inflammatory drugs are also designed to alleviate the side effects of diabetes [22].

**Figure 2:**
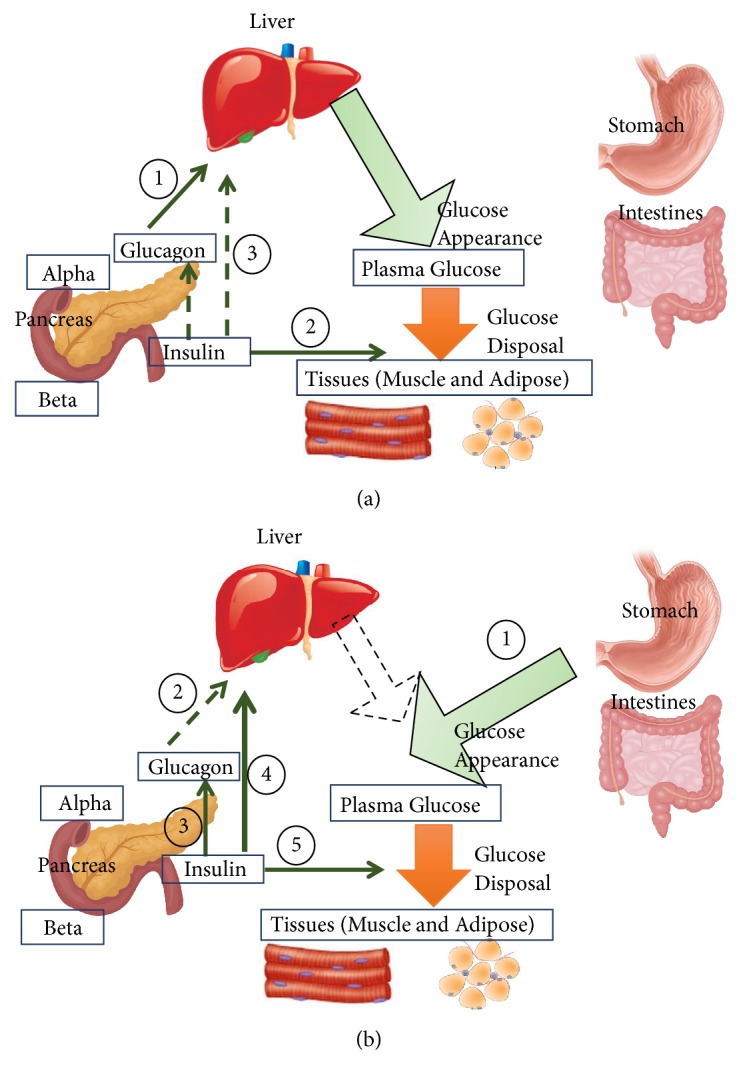
*Glucose homeostasis: role of insulin and glucagon.* In the fasting state (a), serum glucose is derived from glycogenolysis under the regulations of glucagon (1). Insulin controls glucose disposal at its basal levels (2). Since glucose levels are not high, low levels of insulin have minimal role in supressing glucose appearance in the serum (via glycogenolysis and gluconeogenesis) (3). In the fed state (b), glucose in the plasma is derived from nutrition “stomach and intestine” (1). Glucagon secretion and effect are supressed as a result of insulin secretion (2,3). Communication within the islet cells of the pancreas contributes to this inhibition. (4) As a result, gluconeogenesis and glycogenolysis are supressed in the liver. Glucose disposal is activated in peripheral organs (5) [15]. Figure 2 is reproduced from Saad et al. (2017) ([under the Creative Commons Attribution License/public domain)” [14].

The scope of glycemic control at the organ level is introduced in Figure 2. Different organ systems collaborate to maintain serum glucose in the fasting and postprandial states.

Many drugs are nowadays available in the market that are restricted by their pharmacokinetic properties, limited action, and side effects, e.g., biguanides, sulphonylurea. Medicinal plants are used in all known traditional medical systems [3]. Indeed, Herbal-based remedies are still used as the major form of drugs by about 80% of the world's population. These medications are not usually regulated and some would debate that natural ingredients are safe to health. Indeed, any medication, herbal or synthetic, should follow thorough scientific investigation via screening, validation, preclinical and clinical procedures for the test of their efficacy and toxicity levels [14].

Around one quarter of the used drugs at present are of herbal origin and comprise at least one herbal-derived active compound or chemically modified herbal phytochemicals to produce a pharmaceutically active drug. Active phytochemical components can be used as good alternatives in combinatorial drug industry for the production of drugs that target diabetes. In this respect, many plant extracts were screened and have shown antidiabetic effects in animal test models and in clinical studies [14]. Polysaccharides, such as Anoectochilus roxburghii polysaccharides (ARP), Artemisia sphaerocephala Krasch seed polysaccharide (ASKP), Acanthopanax senticosus polysaccharide (ASP), and Coptis chinensis Franch (Ranunculaceae) polysaccharide-1 (CCPW-1), present in orchids, Astaraceae, Siberian ginseng, and the Chinese goldthreadare are used in the treatment of diabetes [23]. Terpenoids (also called isoprenoids) account for more than 40,000 individual compounds of both primary and secondary metabolisms. Terpenoids are specifically present in many herbal plants [24]. Polyphenols are a large set of compounds that include flavonoids, non-ketone polyhydroxy-polyphenols found in black tea, blueberries, citrus, cocoa, peanut and parsley; phenols, found in berries, chili peppers, oregano, sesame seeds and others; tannins, which conjugate to and precipitate proteins, present in berries, chocolate, legumes, nuts, and pomegranates. Alkaloids are nitrogen-containing organic phytochemicals and include morphine, quinine, ephedrine, etc., present in thyme and the Mediterranean saltbush. Saponins have foaming properties, and are found in basil, fenugreek, and the Mediterranean saltbush. Vitamins are also important components of plants and are known for their action as cofactors of central metabolic processes, e.g., biotin, thiamine, folate and niacin [14].

## 3. Strategies for the Glycemic Control

**Figure 3:**
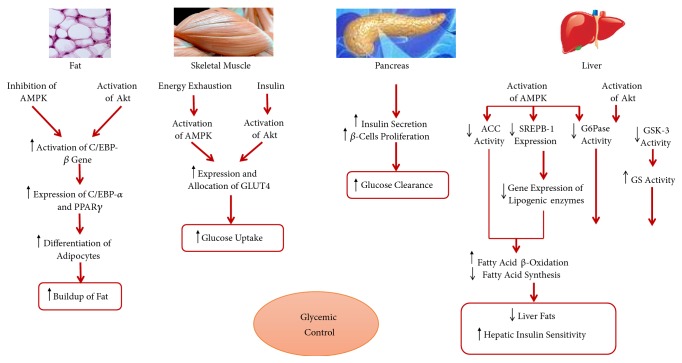
*Contribution of the major tissues to the glycemic control via insulin-dependent Akt and insulin-independent AMPK signaling cascades [14]. *AMPK: AMP-activated protein kinase; Akt: protein kinase B; CEBP: Ccaat-enhancer binding protein; GLUT: glucose transporter; ACC: acetyl-CoA carboxylase; SREPB-1: sterol regulatory element binding protein 1; G6Pase: glucose-6-phosphatase; GSK: glycogen synthase kinase; GS: glycogen synthase. Figure 3 is reproduced from Saad et al. (2017) ([under the Creative Commons Attribution License/public domain)” [14].

Main body organs/tissues that are involved in controlling the homeostasis of serum glucose have direct effect on signaling cascades involved in glycemic control. This relationship is shown in Figure 3.

To achieve serum glucose homeostasis around a “set point” (~5-5.5 mM) [25], balance should be maintained between the rate of glucose entering the bloodstream (glucose appearance) and glucose removed from the circulation (glucose disappearance). Glucose depletion takes place in direct routes such as glycolysis, tricarboxylic acid cycle, and the pentose phosphate pathway [26]. Indirect consumption and regeneration of glucose occur through glycogen and lipid metabolism [25]. The major hormones to regulate plasma glucose levels are insulin (which controls glucose disappearance), glucagon (which regulates glucose appearance in the liver) [27, 28], and epinephrine (regulating glucose appearance in the muscle). Less significant contributions take place by cortisol, norepinephrine, and growth hormones [29]. Alternatively,* local* regulation of glucose levels takes place via allosteric mechanisms, among others.

Two global pathways are key contributors in regulating serum glucose homeostasis. The first introduces insulin-dependent mechanisms for the uptake of glucose and induces protein kinase B (PKB; also known as Akt) signaling [30]. The other signaling pathway is insulin-independent that induces AMP–activated protein kinase (AMPK) signaling cascades [31]. In the insulin-dependent glucose uptake, insulin binds to cell surface receptors, e.g., the insulin receptor-related receptor (IRS) and the insulin-like growth factor (IGF)-I receptor [32]. In the liver tissue, regulation of glucose and fat metabolism is the key effector in maintaining glucose homeostasis. Due to insulin-induced signaling, gluconeogenesis is inhibited; e.g., the activity promoter region of the glucose-6-phosphatase (key enzyme of gluconeogenesis) gene is attenuated and the expression gets reduced [33]. Additionally, glycogen storage is enhanced via dephosphorylating glycogen synthase (GS) and inhibiting glycogen synthase kinase 3 (GSK3), responsible for the phosphorylation of GS; among others [34]. On the other hand, due to AMPK signaling, regulation of fat metabolism at the level of gene expression as well as protein activity follows. Here, the sterol regulatory-element binding protein, SREBP-1 is inhibited and the expression of lipogenic genes is repressed [35]. At the protein level, the activity of acetyl-coA carboxylase is attenuated. As a result, biosynthesis of fatty acid is suppressed and the *β*-oxidation of fatty acids gets stimulated [36]. In skeletal muscles, insulin binding or contraction-induced molecular signaling (via Ca^+2^ and NOS in the proximal region as well as SNARE and Rab-GTPase proteins of the cytoskeleton in the distal region) enhances the expression and translocation of GLUT4 transporters for glucose uptake [37–40]. In adipose tissues, elevated levels of serum glucose induce lipid synthesis and inhibit adipose tissue differentiation. Here, the CCAAT-enhancer binding protein (C/EBP) is enhanced and the gene expression of transcription factors follows, e.g., C/EBP-*α* and the peroxisome proliferation-activity receptor, PPAR- *γ* [41]. Insulin and glucagon are mutually synergistic and act to induce opposite routes of phosphorylating active/inactive states of the key enzymes of sugar metabolism, which aids in shifting the equilibrium towards the directions of glucose clearance or production, respectively [42].

Local control of glucose homeostasis takes place in liver, muscle, and adipose tissues via the allosteric pool of enzymes, proteins, and other macromolecules [32]. Liver tissue has an unparalleled proficiency for “intuiting and buffering” glucose levels in plasma. In this respect, many hepatic isozymes and protein homologues have their singularities in the hepatic tissue. One example is the coded glucose transporters in the liver tissues (GLUT2, found also only in pancreatic and kidney cells, K_m_ = 17-20 mM) controlling glucose influx at high blood glucose concentrations [43]. Additionally, the isozymic form of liver hexokinase (glucokinase) has many folds higher K_m_ to glucose than most other hexokinase isoforms. Moreover, feedback inhibition by the product Glucose-6-phosphate does not happen for the liver isoform, but rather in other tissues [44]. The liver pyruvate kinase activity is noticed at very high plasma levels of glucose [45]. Glucose 6-phosphatase buffers glucose to the blood only from the liver and, to a lesser extent, the kidney [46].

Yet, carbohydrate elimination after a carbohydrates-rich meal is managed by the insulin-sensitive GLUT4 transporters, mainly allocated in muscle tissues and in adipose tissues [47, 48]. Upon exposure to high levels of plasma glucose, muscle tissues store their needs of glycogen [47]. Nonetheless, experiments on knockout mice in muscle glucose transporters showed normal mice in terms of glucose tolerance. Still, knockout experiments on glucose transporters of adipose tissues showed impaired glucose tolerance in muscle, liver, and adipose tissues, which suggests a regulatory role of adipose tissues beyond their glucose absorption capabilities [49].

The kidney, eye, some nerve tissues, erythrocytes, and leukocytes are nearly not responsive to insulin concentration in the blood. Nonetheless, they have a role in glucose depletion for their energy needs, which cannot stand the high levels of glucose in diabetic patients [12].

In summary, the mechanism of action for the antidiabetic activity of plants falls into several routes: increased pancreatic secretion of insulin by the augmentation of the pancreas; inhibition of glucose production in the liver and enhanced glucose uptake in the muscle and adipose tissues; inhibition of glucose absorption by the intestinal; the inhibition of diabetes-related complications. We have studied these mechanisms in detail previously [14]. Diabetes, metabolism disorders, and the involvement of medicinal plants were previously reviewed by us [50]. We summarize below the five aforementioned mechanisms and additionally introduce here the inhibition of epigenetic marks associated with diabetes (see next Section 4).

*Increased pancreatic secretion of insulin-augmentation of the pancreas and increased insulin sensitivity:* Type II DM is characterized by insulin resistance, reduced insulin production, or the failure of pancreatic *β*-cells. Drugs that target the pancreas aim to increase the size of pancreatic islands and the number of cells. Insulin levels can also be augmented through the ATP-dependent K-channels in the pancreatic cells, or drugs that mimic insulin action.

*Inhibition of glucose production in the liver and the enhanced glucose uptake in the muscle and adipose tissues*: Liver is the most crucial organ in regulating serum glucose levels. Liver utilizes the enzymes of glycolysis, gluconeogenesis, and glycogen metabolism to balance blood glucose levels. In addition to the liver tissue, muscle and adipose tissues respond to insulin and increase glucose transporter-4 (GLUT4) in the plasma membrane as a response to insulin secretion (in non-diabetic subjects). As a result, drugs that target liver metabolism and GLUT4 transporters are of interest to pharmacological research.

*Inhibition of glucose absorption:* The inhibition of digestive enzymes that hydrolyze complex polysaccharides and disaccharides into smaller fragments of monosaccharaides is a direct route for the inhibition of their escape to the bloodstream. Such monomer components can be absorbed through the intestinal walls to the bloodstream and absorbed by the liver, muscle, and fat tissues. An example of this group of inhibitors is the mammalian *α*-glucosidase inhibitor.

*Inhibition of diabetes-related complications:* The inflammatory complications of diabetes (e.g., nephropathy, neuropathy, and retinopathy) result from the oxidative damage. Treatment of diabetes alone in most cases does not reverse the disorders, which introduces the need for drugs to alleviate these disease states.

**Figure 4:**
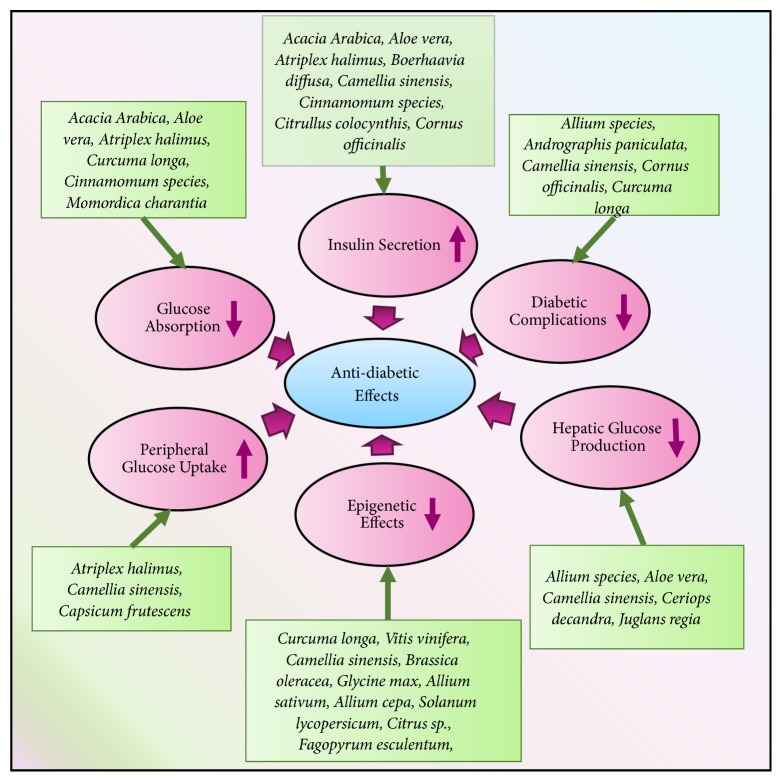
*Antidiabetic medicinal plants and their mechanisms of action of.* Plants work via different modes of action to alleviate diabetes. Some medicinal plants are summarized and their mechanisms of actions are mentioned.

Figure 4 introduces some medicinal plants that work to target diabetes and alleviate the symptoms via the aforementioned mechanisms. For a detailed view on the mechanisms through which plants exert their effects, the readers are directed to [14, 50].

## 4. Epigenetics of Diabetes and Epidrugs

Although there is no uniform definition of epigenetics, it has been described as heritable changes in gene expression and downstream activity that does not target DNA sequence. Epigenetic modifications can be passed from one cell generation to the next (mitotic inheritance) and between generations of a species (meiotic inheritance). Such changes include, for example, DNA methylation, histone methylation, and acetylation. The nucleosome is composed of DNA wrapped around a histone octamer, composed of four histone protein units, two H2A-H2B-dimers, and an H3-H4 tetramer. Between two consecutive nucleosomal cores is a DNA sequence connected with a single molecule of histone H1. Chromatin modifiers at the epigenetic level usually target the amino acids of the* N*-terminal tails of histones and either enable or hamper transcription factors and other DNA binding proteins. At the level of DNA methylation, the 3D structure of chromatin and the minute supercoiling are affected by the methylation status. Changes to the structure of chromatin targets gene expression by either inactivating genes, when the chromatin is closed (heterochromatin), or by activating them when the chromatin is open (euchromatin) [51]. These changes are heritable and regulate gene expression and activity during development and differentiation as well as in response to environmental stimuli, such as nutritional life style. Such changes can nonetheless be also reversible. Thus, they are potential targets for therapeutic drugs.

**Figure 5:**
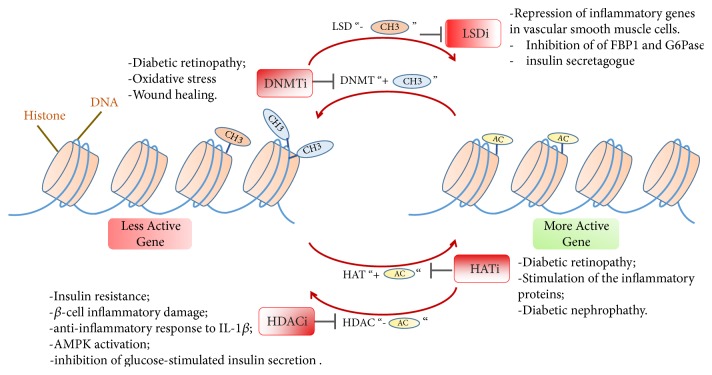
*An overview of the main epigenetic mechanisms and their association with diabetes.* Inhibitors/activators of the several epigenetic marks were shown to alleviate the effects or symptoms of diabetes via several routes. Details for such mechanisms are in the main text. LSDi: lysine-specific demethylase inhibitor; DNMTi: DNA-methyltransferases inhibitor; HATi: histone Acetyltransferase inhibitor; HDACi: histone deacetylase inhibitor; CH_3_: methyl group; AC: acetyl group.

Epigenetic drugs, or “epidrugs”, are an emerging field or class of drugs that target epigenetic changes to treat a wide variety of diseases. These include the metabolic disorders, e.g., diabetes and obesity and a wide variety of many other disorders, including cancer [52] and neurodegenerative diseases [53]. Many epigenetic drugs are now already in the phase of clinical trials for the treatment of diabetes. Some of the targets of epigenetic drugs include DNA methyltransferases, histone-modifying enzymes, e.g., histone deacetylases, histone acetyltransferases, protein arginine methyltransferases, histone methyltransferases, and histone demethylases. Normally, epidrugs serve as inhibitors for such targets, but they can also serve as potential activators in other contexts. Since epidrugs work on the 3D architecture of chromatin, they expectedly target a large network of biological molecules in signaling and metabolic pathways [52]. A brief overview of epigenetic mechanisms, their association with diabetes and the potential epidrugs is introduced in Figure 5.

DNA methylation of cytosine residues takes place at the C-5 position to yield 5-methylcytosine. DNA methyltransferases induce de novo (DNMT3a and DNMT3b) and maintenance (DNMT1) methylation of DNA. This process is accomplished with the aid of an S-adenosyl-methionine, which acts as a methyl donor for methylation [54]. Regulation by DNA methyltransferases has a role in the progression of diabetes, especially in the mitochondrial DNA [55]. One complication of hyperglycemia is the development of diabetic retinopathy, which is suggested to have a metabolic memory phenomenon after hyperglycemia is alleviated [56]. In this respect, DNA is dynamic, but its memory can last for several years. DNA methylation was found to be correlated with the metabolic memory of diabetic retinopathy, where the DNA methyltransferase DNMT1 is highly expressed. In this respect, the introduction of DNMT inhibitors (DNMTi), e.g., Aza, during the reversal period from hyperglycemia, could alleviate the symptoms of retinopathy [57]. Recently, diabetes was found to induce the damage in the wound healing process in a DNMT1-dependent mechanism. This study was applied on hematopoietic stem cells (HSCs) during their differentiation towards macrophages in type II diabetic mice. The process includes a Nox-2-dependent escalation of the oxidant stress in HSCs and a consequent decline in microRNA let-7d-3p. This resulted in the upregulation of DNMT1, which induced hypermethylation of* Notch1*,* PU.1*, and* Klf4*. As a result, the number of wound macrophages drastically decreased [58]. Other DNMTi also exist, e.g., procainamide and hydralazine, which are DNMTi that underwent* in silico *drug prediction and are presently in clinical trials for the treatment of diabetes [59, 60].

Histone deacetylase inhibitors (HDACi) are a new class of drugs. Some of these drugs target genes and proteins associated with diabetes. These drugs were found to efficiently manage insulin resistance in type II diabetes mellitus in preclinical and clinical trials [61]. Some drugs affect *β*-cell functions, prevent *β*-cell inflammatory damage, and relieve insulin resistance. HDACi also induce *β*-cell proliferation and differentiation and might alleviate late diabetic microvascular complications [62]. HDACi show also likely anti-inflammatory properties to IL-1*β* [62]. IL-1*β* is secreted from mononuclear cells and it inhibits *β*-cell function and induces *β*-cell death after prolonged exposure [63]. Among HDACis, valproic acid and sodium phenylbutyrate (PBA) are already in clinical trials for the treatment of diabetes [64, 65]. VPA is an activator of AMP-activated protein kinase (AMPK). Incubation of primary mouse hepatocytes with VPA resulted in higher than normal levels of phosphorylated AMPK and acetyl-CoA carboxylase (ACC), a key enzyme in glucose metabolism. Valproic acid was also found to reduce hepatic fat accumulation, liver mass, and serum glucose in obese mice [66]. Phenylbutyrate (PBA) protects against cardiac injury [65]. PBA also enhances palmitate-induced inhibition of glucose-stimulated insulin secretion [67].

One more class of drugs that treats diabetes targets the histone acetyltransferase (HAT). One such aberrant epigenetic chromatin event that results from hyperglycemia is a significant increase in histone acetylation in retinas from the diabetic rats, suggested to have a metabolic memory [68]. This acetylation was proposed to contribute to the hyperglycemia-induced upregulation of proinflammatory causative proteins for the diabetic retinopathy. To this aim, inhibitors of histone acetyltransferase (garcinol and antisense against the histone acetylase, p300) and activators of histone deacetylase (resveratrol and theophylline) are introduced to lessen both the acetylation and stimulation of the inflammatory proteins [68]. New evidence has shown that HATs and HDACs inhibitors serve also to cure diabetic nephropathy in cellular and animal models [69].

Histone H3 lysine 4 dimethylation (H3K4me2) is a main chromatin mark associated with open chromatin and active gene expression. Lysine-specific demethylase1 (LSD1) regulates H3K4 methylation negatively and reduces its occupancy at gene promoters. Chromatin immunoprecipitation experiments revealed that the promoters of two inflammatory genes, namely, the monocyte chemoattractant protein-1 and interleukin-6, are highly enriched with H3K4me2 in vascular smooth muscle cells (VSMCs) of diabetic mice. Protein levels of LSD1 were, in contrast, drastically diminished [70]. Silencing of LSD1 gene promoted inflammatory gene expression in nondiabetic VSMCs. On the other hand, overexpression of LSD1 in diabetic VSMCs repressed the expression of inflammatory genes [70]. Other studies on HepG2 cells presented the inclusion of LSD1 in the activation of gluconeogenesis pathways, thus leading to an increase in serum glucose levels and a decrease in intracellular glycogen. LSD1 was found to allocate in the promoters of FBP1 and G6Pase, two key enzymes of gluconeogenesis, and to regulate their H3K4 dimethylation levels [71]. Thus, drugs that target LSD1 must be targeted in tissue- and context-specific manners. Tranylcypromine is an FDA-approved drug used to treat major depressive disorder. It is now recognized as a histone demethylase inhibitor of lysine-specific demethylases (LSD1 and LSD2). Tranylcypromine was described as an effective insulin secretagogue and hypoglycemic agent [72].

### 4.1. Herbal-Derived Anti-Diabetes Epidrugs

Recently, natural compounds, such as resveratrol, curcumin, and epigallocatechin gallate (EGCG), have been shown to alter epigenetic mechanisms, which may lead to the increased sensitivity of cancer cells to conventional agents and the inhibition of tumor growth [73].

Resveratrol is a natural polyphenol found in grapes and chocolate. Over a decade, resveratrol was found to activate sirtuin 1 (SIRT1), an NAD-dependent HDAC whose administration to insulin-resistant animals improves glucose homeostasis and regulates insulin sensitivity [74–76]. Until recently, however, only few clinical trials exist that have tested the health benefits of resveratrol in humans with metabolic deficiency [77]. Curcumin is an inhibitor of HATs, HDACs, and DNMTs. It also serves as inhibitor or activator of several miRNAs [78]. Epigallocatechin gallate (EGCG) is the most abundant green tea catechin. Epigenetic mechanism of action for this drug involves histone acetylation-deacetylation and DNA methylation, where EGCG upregulates the anti-inflammatory activity of regulatory T cells [79].

Other polyphenols also exist that have epigenetic targets. One such example is sulforaphane of broccoli, which is an epigenetic drug that was found to inhibit DNMT1 expression, reduce promoter methylation, and inhibit HDACs [80–82]. Cell culture,* in vivo* studies, and analysis of coexpression networks and genetic data of the liver tissue showed that sulforaphane can inhibit glucose production through mechanisms of nuclear translocation of nuclear factor erythroid 2-related factor 2 (NRF2) and the inhibition of gene expression of crucial enzymes of gluconeogenesis [83]. Genistein is a polyphenol of soy beans which reverses hypermethylation and induces active histone modifications in many tumors [84]. Genistein seems to modulate on diabetes via direct effects on *β*-cell proliferation, glucose-stimulated insulin secretion, and protection against apoptosis. This is suggested to involve cAMP/PKA signaling cascades and to modulate via epigenetic mechanisms [85]. Organosulfur compounds of garlic and allium also have anti-diabetic effects. These natural products were also found to modulate via the induction of histone acetylation in several malignancies [86]. Lycopene is a phytochemical present in tomatoes with a potent antioxidant effect. Some studies expected a beneficial outcome in using this phytochemical to relieve the oxidative stress of diabetic patients [87]. This drug was found to function via gene methylation modes [88]. Quercetin is another epidrug present in citrus fruits and buckwheat. This drug acts as a DNMT1 inhibitor (via the repression of TNF-induced NFkappa transcription factor) and promotes Fas ligand-related apoptosis via histone H3 acetylation and potential HDAC inhibition [89–91]. Quercetin was shown to be involved in the stimulation of glucose uptake through MAPK insulin-dependent mechanism. This is accomplished in the muscle via the translocation of GLUT4 transporters and in the liver via the downregulation of key gluconeogenesis enzymes [92, 93].

## 5. Protein Targets for the Treatment of Diabetes

**Figure 6:**
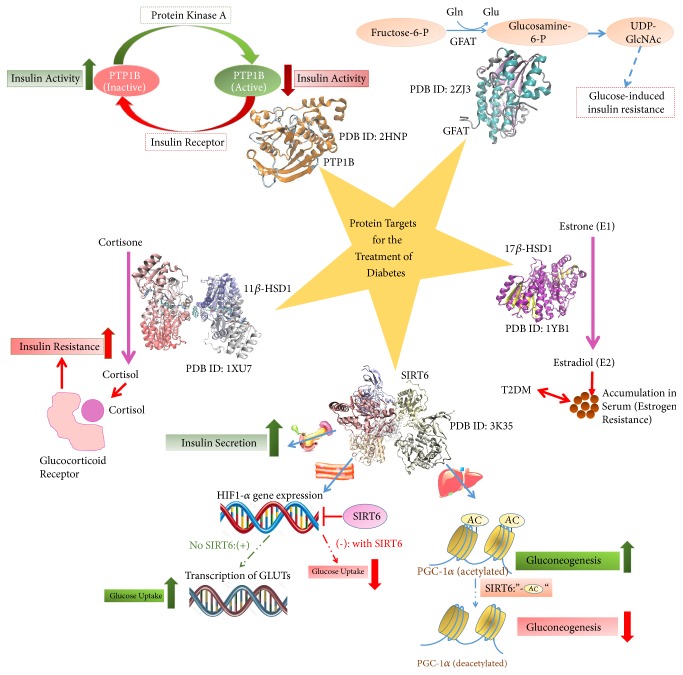
*Protein targets for antidiabetic drugs and their mechanisms of action.* Details for the targeted effect on these enzymes by antidiabetic phytochemicals are discussed in the main text. PDB: protein data bank file; PTP1B: protein tyrosine phosphatase 1B; GFAT: glutamine:fructose-6-phosphate aminotransferase; GlcNAc: N-acetyl-glucosamine; 17*β*-HSD1: 17*β*-hydroxysteroid dehydrogenase 1; 11*β*-HSD1: 11*β*-hydroxysteroid dehydrogenase 1; T2DM: type II diabetes mellitus; SIRT6: sirtuin 6; GLUTs: glucose transporters; HIF1-*α*: hypoxia-inducible factor 1-alpha.

Computer-aided drug design is nowadays used to screen the phytochemical “lead compounds” which can be antidiabetic. Quantitative structure-activity/property relationships help us filer drugs that can be administered to the human biological system with high efficiency and less side effects. Nonetheless, once the feasible phytochemicals are selected, the modes of interaction with the biological targets aid in establishing the decision for their effectiveness as antidiabetic agents. Docking studies, molecular dynamic simulations, and free energy calculations predict the detailed picture for the action mechanisms as well as interactions involved between the lead and target in the process of drug design. Below, we list the common protein targets for the treatment of diabetes and discuss some phytochemicals that are expected to directly affect the activity of these targets.  Figure 6 introduces the link between these protein targets and diabetes. Additionally, phytochemicals that target these proteins are briefed.

### 5.1. 11*β*-Hydroxysteroid Dehydrogenase

11*β*-hydroxysteroid dehydrogenase (11*β*-HSDs) is a member of the short-chain reductases (SDR). It catalyzes the interconversion between the active glucocorticoids (corticosterone, cortisol) and the inert 11-keto forms (cortisone, 11-dehydrocorticosterone). 11*β*-HSD has several isoforms in humans, which are available in the liver, brain, adipocytes, lung, and other tissues. The 11*β*-HSD1 is NADPH-dependent active isoform, which is mainly expressed in the liver as well as the adipose tissue [94]. It is responsible for maintaining a sufficient exposure of relatively low affinity glucocorticoid receptors to their ligand [95]. The 11*β*-HSD1 faces the ER lumen, and this compartmentalization is crucial for its regulation. The cofactor NADPH that is regenerated by hexose-6-phosphate dehydrogenase is also located in the ER lumen [96].

Cortisol plays a pivotal role in diabetes. Indeed, the abnormal regulation of glucocorticoid metabolism was linked to type II diabetes [97]. The antagonism of hepatic glucocorticoid receptor was found to reduce glucose levels in the serum of diabetic mice and to ameliorate insulin resistance [98]. Thus, 11*β*-HSD is a potent therapeutic target whose inhibition might serve in the treatment of type II diabetes [99, 100].

The crystal structure of 11*β*-HSD1 was first resolved in 2005 for* Mus musculus *[101]. There exist so far 11 structures for the human isozyme, two murine structures, and a single structure for guinea pig in the RCSB database [102]. Hosfield and colleagues provided the structure of human 11*β*-HSD1 in both the open and closed conformations [103]. 11*β*-HSD1 is a tetramer composed of two dimers. The overall topology of 11*β*-HSD1 includes, as in other SDR enzymes, central 6-stranded, all-parallel *β*-sheets that are sandwiched by three *α*-helices. The active site is found in the region where the NADP+ and the steroidal detergent CHAPS molecules are located. The substrate binding induces the reorientation of the variable *β*6-*α*6 insert that is specific to 11*β*-HSD1 in order to provide the hydrophobic interface needed for binding and exclude the bulk solvent. The center of the tetramer has a Pro-Cys motif, which forms reversible disulfide bridges that can change the enzyme activity. Structural changes at the enzyme active site were found to be coupled with conformational flexibility at the tetramerization interface, which suggests a mechanism for the enzymatic activity [103]. This motif is located at the C-terminus and caps the active site of the next subunit in the tetramer. On the other hand, the N-terminal portion is responsible for orienting the enzyme in the ER membrane [104]. Of the crystal structures resolved for the human protein, several studies served to investigate the binding of 11*β*-HSD to its inhibitors, such as sulfonamide and triazole, which showed competitive and mixed inhibitions, respectively. Triazole interacts closely to the NADP+ cofactor and is assumed to change the NADP+ binding [105].

Hintzpeter and colleagues investigated the plausible inhibition of 11*β*-HSD1-mediated cortisone reduction upon the introduction of dried leaves of green tea to human microsomes, which turned out to be positive [106]. Subsequently, polyphenolic compounds were extracted from green tea and tested. Amongst the phytochemicals in green tea, (-)-epigallocatechin gallate (EGCG) exhibited the strongest inhibition of 11*β*-HSD1 (IC50* *=* *57.99 *μ*M for reduction; IC50* *=* *131.2 *μ*M for oxidation). Competitive inhibition was proposed to be the mode of action for EGCC. Docking studies showed the allocation of EGCG in the active site of 11*β*-HSD1, where it hydrogen bonds with Lys187 [106]. Ginger is also known for its anti-diabetic activity. Three gingerol derivative compounds are [6]-paradol, (E)-[6]-Shogaol, and (5R)-Acetoxy-[6]-Gingerol shown, known for their inhibitory activities against human and mouse 11*β*-HSD1 [107].

In a study by Tsang and colleagues [108], the administration of pomegranate juice to human volunteers was tested for its possible inhibition of 11*β*-hydroxysteroid dehydrogenase type 1. The volunteers consumed 500 ml of pomegranate juice, and a negative control group consumed 500 ml of a placebo drink containing the same levels of energy for 4 weeks. Measurements were performed for cortisol/cortisone ratio in the urine, and it was to be significantly lowered in the group of volunteers who have taken pomegranate juice for the whole time when compared to the placebo control [108].

Licorice is another plant that was also found to inhibit 11*β*-hydroxysteroid dehydrogenase type 1 [109]. Gumy and colleagues investigated the effect of the leave extracts of loquat in transfected HEK-293 cells and found that the extracts were capable of inhibiting 11*β*-HSD1. Additionally, extracts of roasted but not native coffee beans were found to exhibit the same effects [110].

### 5.2. 17*β*-Hydroxysteroid Dehydrogenase

17*β*-hydroxysteroid dehydrogenases (17*β*-HSDs) play key roles in the first step of the degradation of androgen and estrogen as well as the last step in their activation. The weaker estrone (E1) can be synthesized from the more potent estrogen, estradiol (E2) by oxidative 17*β*-HSDs [111]. 17*β*-HSD2 catalyzes the production of E1 using NAD as a cofactor (Km =0.35±0.09 *μ*M) [112]. Conversely, E1 can be converted to E2 by reductive 17*β*-HSDs, including the highly active 17*β*-hydroxysteroid dehydrogenase 1 (17*β*-HSD1). The 17*β*-HSD1 isoform has a higher specific activity than the 17*β*-HSD2 enzyme [113]. The 17*β*-HSD1 has a dual function, as it is also slightly involved in catalyzing the conversion of active androgens, such as 4-androstenedione, to inactive ones, e.g., testosterone [114]. 17*β*-HSD1 performs its catalytic activity with the help of NADPH as a cofactor (Km value of 0.03±0.01 *μ*M) [115].

In humans, 17*β*-HSD1 is expressed in endometrium, ovary, placenta, and breast. Indeed, the enzyme was found to be more profusely expressed than 17*β*-HSD2 in estrogen-dependent breast cancer cells [116, 117]. Additionally, Zhang and colleagues [118] showed that the expression of 17*β*-HSD1 is crucial in determining the [E2]/[E1] ratio in breast cancer cells. E2 was found to induce metabolic homeostasis. Thus, accumulation of the circulating E2 in serum might be indicative of estrogen resistance. This indeed might be linked to metabolic deficiency and T2DM [119]. As a result, 17*β*-HSD1 might provide a good therapeutic target for inhibitory drugs for the treatment of diabetes. Nonetheless, most inhibitors of this steroid-converting enzyme are estrogen analogues, which poses an obstacle in the clearance of their estrogenic activities.

Amongst the human steroid-converting enzymes, the crystal structure of 17*β*-HSD1 was the first to be resolved. The enzyme was crystallized in the presence of NADP-, *β*-octylglucoside, glycerol, and polyethylene glycol. Unlike other structures of the short-chain dehydrogenases (SDRs), this reductase enzyme was found to have an insertion of two helix-turn-helix motifs, suggested to contribute to the substrate specificity and membrane integration [120]. 17*β*-HSD1 is 327-amino acid long and exists as a homodimer [121, 122]. Recombinant human 17*β*-HSD1 in complex with estradiol were captured at room temperature, and the structure was resolved at 1.7 Å [123]. The enzyme-estradiol interactions include a hydrophobic core of the steroid and nine residues in the enzyme binding pocket as well as hydrogen bonds, which contribute to the enzyme specificity. The three hydrogen bonds involve the side chains of Ser142, Tyr155, and His221. Additionally, Glu282 contributes to the binding process at the interface. C-19 steroids bind to 17*β*-HSD1 in both normal and reverse orientations, which induces an inhibition of the most potent androgen dihydrotestosterone (DHT). The mechanism involves the 3*β*-reduction of DHT into 5-androstane-3,17-diol (3*β*-diol) and 17*β*-oxidation of DHT into A-dione [124].

The structure of the ternary complex of 17*β*-HSD1 with the cofactor NADP^+^ and equilin (an estrogen used in estrogen replacement therapy, 3.0-Å resolution) was solved by Sawicki and colleagues [125]. Equilin was found to inhibit the 17*β*-HSD1-catalyzed reduction of E_1_ to E_2_, and the crystal structure showed that the equilin molecule is bound at the active site in a similar fashion to the substrate [125].

Complex phenols in olive oil were investigated for their inhibitory action on both reductive and oxidative 17beta-hydroxysteroid dehydrogenase activity in human hepatic microsomes. Dihydroxybenzoic acid, gallic acid, hydroxytyrosol, and oleuropein glycoside could inhibit the reductive 17beta-HSD activity but not the oxidative one. Rather, gallic acid stimulated the activity by approximately 30% [126].

### 5.3. Glutamine Fructose-6-Phosphate Amidotransferase (GFAT)

Glutamine-F-6-P amidotransferase (GFAT) is an enzyme that shifts the flow of the incoming glucose into the hexosamine biosynthesis pathway. This pathway is a minor branch in the glycolysis pathway; it is nonetheless crucial for the glycosylation of proteins and lipids. GFAT is a rate-limiting enzyme that converts fructose-6-phosphate to glucosamine-6-phosphate. First, acetyl-coenzyme (CoA) is derived from either glucose metabolism or fatty acid *β*-oxidation, and it transfers its acetyl group to glucosamine-6-phosphate to yield N-acetylglucosamine-6-phosphate [127, 128]. In a second step, the main end-product of the pathway, UDP-N-acetylglucosamine (UDP-GlcNAc), is produced, where a uridine nucleotide (UDP) is added to the glucosamine. UDP-GlcNAc is employed in N- and O-linked glycosylation. Glucose-induced insulin resistance is a possible consequence of this shift to the hexosamine pathway and the resultant glucose toxicity [129–131]. Crystal structures of GFAT with UDP-GlcNAc could offer strategies to derive lead compounds that target type 2 diabetes for treatment.

To help investigate the changes in enzymatic activity of GFAT, Traxinger and Marshall [132] found that the treatment of isolated rat adipocytes with insulin or glucose alone (or in combination) failed to reduce cytosolic GFAT activity after 4 h treatment. The combined treatment with insulin, glucose, and glutamine altogether caused a dramatic loss (70%) of GFAT activity in less than 2 h. Extensive treatment of the adipocytes with glucosamine (360 *μ*M), which is a part of the hexosamine pathway, elicited a 55% loss of GFAT activity after 4 hours [132].

Glutamine analogs were used to assess the role of glutamine in the expression of glucose-induced desensitization of the insulin-responsive glucose transport systems (GTS). 0-diazoacetyl-L-serine (azaserine) and 6-diazo-5-0~0- norleucine are glutamine analogs that could irreversibly inactivate glutamine-requiring enzymes, such as glutamine:fructose-6-phosphate amidotransferase (GFAT). Both azaserine and 6-diazo-5-0~0- norleucine were found to inhibit the desensitization in 18-h treated cells without affecting maximal insulin responsiveness in control cells [132].

Human GFAT enzyme exists in two isoforms and a splice variant, GFAT1, GFAT2, and GFAT1L [133, 134]. GFAT1 was found to be highly expressed in striated muscles and adipose tissues, and to a lesser extent in the liver [135–137], which are major targets for the treatment of diabetes and obesity. GFAT has two distinct domains. The first is an N‐terminal glutaminase domain (27 kDa), responsible for converting glutamine to glutamate and ammonia. The other domain is a C‐terminal isomerase domain (40 kDa), which includes the active site that exploits ammonia to transform fructose-6P into glucosamine-6P [138]. Nakaishi and colleagues resolved the first crystal structure of human isomerase domain of GFAT ([139] PDB ID: 2ZJ3). The conformation of the active site is rigid, and it is composed of two analogous subdomains (residues 313–493 and 494–508). Each subdomain is composed of five parallel *β* sheets surrounded by *α* helices.

In a study by Shetty and Salimath, diabetic mice that were fed with starch diet exhibited an increase in the activity of GFAT when compared to the control group. The addition of fenugreek dramatically controlled this increase in GFAT activity, which indicates the inhibitory activity of fenugreek phytochemicals against GFAT [140].

The active phytochemical linalool in* C. sativum* was docked in one study to the protein GFAT (PDB: 2JZ3) [141]. The docking studies showed van der Waal interaction with Ala674, Cys373, Thr425, Gly374, Ser376, Thr375, Ser473, Ser676, Val471, Lys675, and Glu560 [141].

### 5.4. Protein Tyrosine Phosphatase 1B (PTP1B)

The covalent addition of a phosphate (PO_4_) group by kinase enzymes into a protein is a posttranslational modification that is biologically significant. It is involved in regulating metabolic and signal transduction processes. This modification results in the downstream inhibition or activation of the target receptor proteins and enzymes. In eukaryotes, serine, threonine, and tyrosine are the amino acids normally targeted for phosphorylation [142]. Ushiro and Cohen identified tyrosine phosphorylation as a result of epidermal growth factor-activated protein kinase [143]. The process of tyrosine phosphorylation is involved in cell proliferation and differentiation [144, 145]. Phosphorylation and dephosphorylation of tyrosine are mediated via protein tyrosine kinases (PTKs) and protein tyrosine phosphatases (PTPs), respectively. Binding of type 1 insulin-like growth factor (IGF-1) to its tetrameric receptor induces auto-phosphorylation of the receptor and the downstream activation of PKB and MAPK pathways [146, 147]. This indeed is involved in the translocation of GLUT4 transporter to the plasma membrane. PTPs constitute a huge family of enzymes with a conserved 11-residue sequence motif of PTPs and dual specificity phosphatases. Arginine and cysteine in this motif are indispensable for the recognition and catalytic removal of phosphates. PTP1B is the first isolated PTP, which is involved in regulation the insulin-signaling pathway [148]. Barfold and colleagues resolved the first crystal structure of PTP1B in 1994 [149] with 321 residues. In this structure, the phosphate recognition domain is located in a loop at the N-terminal side. On the other hand, the catalytic site is located at the base of a shallow cleft. The cysteine is involved as a nucleophile in the cleavage.

Inhibitors of PTP1B are designed to alleviate type 2 diabetes mellitus and manage insulin resistance. These inhibitors were found to stably bind in the two binding sites and show their effect at nanomolar concentrations [148]. Nonetheless, searching for inhibitors that bind in the active site might be not the most effective as the positively charged active site is highly conserved among the proteins in the family of tyrosine phosphatases. As such, a better alternative strategy is to search for an allosteric site for inhibition. Jin and colleagues [150] used molecular docking, binding free energy calculations, and molecular dynamics simulations, and they found an allosteric site that is less conserved and more hydrophobic. The inhibitor used in their study was lupane triterpenes, which proved to inhibit PTP1B in cell culture studies. The computational studies were followed by two enzymatic assays for validation [150].

Jiang and colleagues [151] reviewed the natural products that possess inhibitory activities against PTP1B. Thus, this protein seems to be a hot target in phytochemical screening. Phytochemicals that target PTP1B include phenolics, terpenes, steroids, N- or S- containing compounds, and miscellaneous phytochemicals [151].

*Papaver somniferum-derived *papaverine is a member of isoquinoline alkaloids that has a high structural similarity to berberine, a known inhibitor PTP1B in human. Docking studies on papaverine showed a low energy orientation and a good fit in the binding pocket of PTP1B. This was strengthened by* in vitro* studies that show inhibitory action against PTP1B as well as* in vivo* studies that showed a significant reduction in fasting glucose levels in diabetic mice upon the administration of this phytochemical [152].

### 5.5. Mono-ADP-Ribosyltransferase-Sirtuin-6 (SIRT6)

As we previously stated, SIRTs are targeted by anti-diabetes epidrugs. SIRT6 has NAD^+^-dependent deacetylase activity [153] as well as mono-ADP-ribosyltransfease activity [154]. Drugs that target SIRT6 were found to exhibit either inhibitory or activating mechanisms. On one hand, the absence of SIRT6 was shown to be concomitant with increased tissue glucose uptake and decreased serum glucose levels [155]. SIRT6 was proposed to perform its epigenetic effect via inhibiting the expression of the transcription factor hypoxia inducible factor-1*α* (HIF1-*α*), which is involved in the transcription of glucose transporters [156]. Thus, inhibitors were used that target SIRT6 leading to the decreased uptake of glucose by peripheral tissues. On the other hand, SIRT6 was suggested to perform its epigenetic action via the deacetylation of peroxisome proliferator-activated receptor-*γ* coactivator 1*α* (PGC-1*α*), which robustly stimulates glucose production in the liver. Thus, activators of SIRT6 were designed that help repress hepatic gluconeogenesis and decrease blood glucose levels [157].

The resolved structure of SIRT6 showed variation from the structures of other sirtuin proteins in the class. The structures showed an extended zinc-binding domain. Additionally, the helix set that connects the Rossmann fold domain to the zinc-binding motif is absent in SIRT6. Furthermore, due to the absence of the conserved NAD(+)-binding loop, SIRT6 is suggested to be exclusively able to bind NAD(+) in the absence of an acetylated substrate. Indeed, kinetic studies (K(d) = 27±1 *μ*M) proved this hypothesis [158].

Le [159] has performed docking studies on the six main components of ginger ([4]-gingerol, [6]-gingerol, [8]-gingerol, [10]-gingerol, [6]-shogaol, and *β*- bisabolol) with all of the above-mentioned protein targets. *β*-bisabolol and 10-gingerol were found to have low activity and a high metabolization rate. He concluded that the effect of the phytochemicals in ginger is rather synergistic; thus the mixture of all components should be administered as plausible drugs [159].

The effect of* Euphorbia thymifolia* Linn. extracts on mice models was studied, and the plant was found to induce antihyperglycemic effects. To investigate the action mechanism and molecular interactions existing between the bioactive phytochemicals in* E. thymifolia* and protein targets of Type 2 DM, molecular docking, and bond scanning were performed on the interaction between 20 ligands and four of the above target proteins: 11-*β* HSD1, GFAT, PTP1B, and SIRT6. In the next step. Energy calculations indicated strong affinity (<−8.0 kcal/mol) of seven lead compounds to the targeted proteins. Additionally, the molecules had hydrophobic interactions and hydrogen bonds with the active site of the four target proteins. The bioactive phytochemicals are *β*-amyrine, corilagin, cosmosiin, quercetin-3-galactoside, quercitrin, taraxerol, 1-*O*-and galloyl-*β*-d-glucose [160].

## 6. Conclusion

Phytochemicals are hot plausible drugs that are thoroughly investigated for their antidiabetic effects. Studies on signaling cascades and local pool of enzymes and proteins showed the phytochemicals can target such routes to alleviate high serum glucose levels. The current perspective for the effect exerted by phytochemicals to treat diabetes suggests epigenetic modulation, where phytochemicals target central epigenetic marks. Recent interest in epigenetics has focused on phytochemicals aimed at modifying diabetes-specific gene/protein expression. Several major classes of epigenetic agents include drugs/phytochemicals already in the marketplace as well as several in various stages of preclinical as well in clinical investigations. These classes include HDACi, HATi, PRMTis, DNMTis, HDMis, and SIRTis. In this review, we discuss drugs/phytochemicals with epigenetic properties that have been identified as potential therapeutic agents in the treatment of diabetes. Further modeling and cheminformatics studies are current topics in drug discovery applied in drug design for diabetes, and it is expected that the scope for the treatment of diabetes will be transferred into a new era.

## Abbreviations

11*β*-HSD1: 11*β*-Hydroxysteroid dehydrogenase 1
17*β*-HSD1: 17*β*-Hydroxysteroid dehydrogenase 1
AC: Acetyl group
ACC: Acetyl-CoA carboxylase
Akt: Protein Kinase B
AMPK: AMP-activated protein kinase
ARP: Anoectochilus roxburghii polysaccharides
ASKP: Artemisia sphaerocephala Krasch seed polysaccharide
ASP: Acanthopanax senticosus polysaccharide
CCPW-1: Coptis chinensis Franch (Ranunculaceae) polysaccharide-1
CEBP: Ccaat-enhancer binding protein
CH3: Methyl group
CoA: Acetyl-coenzyme
DM: Diabetes mellitus
DNMT: DNA methyltransferase
DNMTi: DNA-methyltransferases inhibitor
EGCG: Epigallocatechin gallate
ER: Endoplasmic reticulum
FBP1: Fructose bisphosphatase 1
G6Pase: Glucose-6-phosphatase
G6Pase: Glucose-6-phosphatase
GFAT: Glutamine:fructose-6-phosphate aminotransferase
GlcNAc: N-Acetyl-glucosamine
GLUT: Glucose transporter
GLUT: Glucose transporter
GLUTs: Glucose transporters
GS: Glycogen synthase
GS: Glycogen synthase
GSK: Glycogen synthase kinase
GSK3: Glycogen synthase kinase 3
GTS: Glucose transport systems
H3K4me2: Histone H3 lysine 4 dimethylation
HATi: Histone Acetyltransferase inhibitor
HDACi: Histone deacetylase inhibitor
HDL: High-density lipoprotein
HIF1-*α*: Hypoxia-inducible factor 1-alpha
HSCs: Hematopoietic stem cells
IGF: Insulin-like growth factor
IRS: Insulin receptor-related receptor
LDL: Low-density Lipoprotein
LSD1: Lysine-specific demethylase1
LSDi: Lysine-specific demethylase inhibitor
NRF2: Nuclear factor erythroid 2-related factor 2
PBA: Phenylbutyrate
PDB: Protein data bank file
PGC-1*α*: Peroxisome proliferator-activated receptor-*γ* coactivator 1*α*
PKB: Protein kinase B
PTKs: Protein tyrosine kinases
PTP1B: Protein tyrosine phosphatase 1B
PTPs: Protein tyrosine phosphatases
SDR: Short-chain reductases
SIRT1: Sirtuin 1
SREPB-1: Sterol regulatory element binding protein 1
T2DM: Type II Diabetes Mellitus
UDP: Uridine nucleotide
VSMC: Vascular smooth muscle cells
WHO: World Health Organization.
